# From chemical shift data through prediction to assignment and NMR LIMS - multiple functionalities of nmrshiftdb2

**DOI:** 10.1186/1758-2946-4-S1-P52

**Published:** 2012-05-01

**Authors:** Stefan Kuhn, Nils E Schlörer, Heinz Kolshorn, Raphael Stoll

**Affiliations:** 1 Aureliusstr. 45, 52067 Aachen, Germany; 2 Department of Chemistry, University of Cologne, 50939 Köln, Germany; 3 Institut für Organische Chemie, Johannes-Gutenberg-Universität, 55128 Mainz, Germany; 4 Biomolecular NMR, Ruhr-Universität Bochum, Bochum, Germany

## 

nmrshiftdb2 and its predecessor NMRShiftDB [1,2] have been available as a community-based NMR database since 2002. During that time a continuously growing set of currently more than 40000 structures with 48600 spectra could be established. These data are freely available (http://nmrshiftdb.org) and cannot only be searched but can also be downloaded and used for scientific investigations. Supplementary to the database, supplementary software was developed including an NMR lab administration system.

Recently, there were some changes in the team and we had a rebranding to nmrshiftdb2. Now, we would be very interested to enter discussion about the project and its future perspectives by receiving feedback from former, current and potential users of different areas. We will discuss the state of this project, e.g. software functions, data collection, and use for research.

An important new feature is the laboratory information management system (LIMS) which has been developed with the intention to better integrate the database functionality into an academic NMR laboratory environment. Nmrshiftdb2 now allows NMR laboratories at the same time to administer and account for their users, instruments and measurements and use the known database functions from the same surface.
